# Off-Label Use of Dalbavancin for Sequential Treatment of Spondylodiscitis by Methicillin-Resistant *Staphylococcus aureus*: A Retrospective Single-Centre Experience

**DOI:** 10.3390/antibiotics11101377

**Published:** 2022-10-08

**Authors:** Maria Mazzitelli, Milo Gatti, Vincenzo Scaglione, Daniele Mengato, Marco Trevenzoli, Andrea Sattin, Federico Pea, Anna Maria Cattelan

**Affiliations:** 1 Infectious and Tropical Diseases Unit, Padua University Hospital, Via Giustiniani, 35128 Padua, Italy; 2 Department of Medical and Surgical Sciences, Alma Mater Studiorum, University of Bologna, 40138 Bologna, Italy; 3 SSD Clinical Pharmacology, IRCSS Azienda Ospedaliero-Universitaria Sant’Orsola di Bologna, 40138 Bologna, Italy; 4 Pharmacy Unit, Padua University Hospital, 35100 Padua, Italy

**Keywords:** dalbavancin, MRSA, spondylodiscitis, off-label, vertebral infections, TDM

## Abstract

**Background:** Our aim was to describe the clinical outcome and safety of the sequential treatment with off-label dalbavancin in patients with spondylodiscitis that is caused by methicillin-resistant *Staphylococcus aureus* (MRSA). **Methods**: We retrospectively included all patients >18 years of age with spondylodiscitis that is caused by MRSA that was treated with dalbavancin from January 2018–January 2021, recording the instances of clinical cure/failure, adverse events, and the need to be re-hospitalized after the initiation of dalbavancin. In 2/15 patients, we performed therapeutic drug monitoring (TDM) for dalbavancin. **Results**: We included 15 patients, 53.3% of them were females, with a median age of 67.9 years (57.4–78.5); 100% patients reported back pain, while a fever was present only in 2/15 cases. The spondylodiscitis was localized in 86.6% cases at the lumbar level. A median of a 2-week in-hospital intravenous vancomycin was followed by dalbavancin with a median duration of 12 weeks (12–16). All patients reported a clinical cure, except for a woman who is still on a suppressive treatment. No patient needed to be re-hospitalized, access to emergency department, or experienced adverse events. The TDM for dalbavancin showed that more than 90% of the determinations were above the pharmacodynamic target against staphylococci. **Conclusions:** The results from our unique, even if it was small, cohort demonstrated that dalbavancin can be a safe/effective option as a sequential treatment in patients with serious infections requiring prolonged antibiotic therapy, such as spondylodiscitis.

## 1. Introduction

Dalbavancin is a novel long-acting lipoglycopeptide antibiotic drug, and it was approved by both the Food and Drug Administration (May 2014) and the European Medicine Agency (February 2015) for the treatment of patients with acute bacterial skin and skin structure infections (ABSSSis) that are caused by Gram-positive microorganisms, including methicillin-resistant *Staphylococcus aureus* (MRSA) [1,2,3]. It is characterized by a favourable pharmacokinetic profile, consisting in a terminal half-life of approximately 14.4 days, a high capacity of plasma–protein binding (93%), a predominant non-renal clearance, and a good tissue penetration [4]. Usually, it is administered by the intravenous route at the dosing of 1000 mg which is followed by 500 mg one week apart from the first one, or 1500 mg in a single infusion [4]. Soon after dalbavancin approval, a growing number of reports described its off-label use as consolidation therapy in acute infections that typically require a long course of antibiotic therapy such as deep-seated infections including endocarditis, other endovascular infections, and bone infections [5,6,7,8,9]. In this setting, dalbavancin use has been shown to be effective and safe, and it has been associated with a decreased length of hospital stay, thereby resulting in an improvement in the patient quality of life, and in an overall cost-saving for the health-care systems [10,11].

Bone infections and spondylodiscitis are difficult-to-treat infections, requiring a long course of antibiotic treatment that needs to be exclusively performed in an in-hospital setting [12,13,14]. Notably, the incidence of spondylodiscitis is constantly increasing for the improvements of diagnostic and treatment surgical procedures [15]. The risk factors for such infections are advanced age, previous infections, the use of drugs by intravenous use, prolonged immunosuppression (either by induced by diseases or iatrogenic), diabetes, transplants, and malignancies [14]. The most common presentation is back pain in more than 90% of patients, while a fever is present just in 35% of cases [16]. In most cases, spondylodiscitis is a monomicrobial infection caused by Gram-positive agents such as *Staphylococcus aureus* (either methicillin-sensitive or resistant strain) which is isolated in more than 50% of cases, followed by coagulase-negative *Staphylococci* (CoNS), *Streptococcus* species, and Gram-negative pathogens such as *Escherichia coli* (11–25% of cases) [15,17,18].

Even though the prognosis for such infections is improving over time, the disease is still challenging with a percentage of complications (such as chronic pain and neurological disability) which ranges from 16 to 32% cases, and a significant impact on the quality of life [19]. An appropriate antibiotic therapy represents a cornerstone in the outcome of the spondylodiscitis. Guideline recommendations on antibiotic treatment are depending on the microbiological isolate [14]. Vancomycin is considered the first-line antibiotic option for infections caused by MRSA [14]. However, it needs to be given intravenously on a twice daily basis (thus, requiring an indwelling catheter) for at least 6 weeks of the treatment, its serum concentrations must be carefully monitored, and its dose has to be adjusted accordingly to the renal function to prevent nephrotoxicity [20,21]. By contrast, dalbavancin is significantly less toxic than vancomycin, it can be infused through a peripheric venous access at the dosage of 1500 mg in single dose, which can be possibly and easily repeated [22,23]. In addition, pharmacokinetic studies demonstrated that this new drug could reach an optimal concentration both in the bone and articular tissues [23]. Real-life dalbavancin treatment for osteomyelitis and prosthetic joint infections has been shown to be effective and safe [24,25]. However, to date, not many clinical data are available for spondylodiscitis [26,27]. Additionally, a proof-of-concept study recently highlighted the clinical usefulness that therapeutic drug monitoring (TDM) may have in estimating the duration of dalbavancin optimal target attainments in osteoarticular staphylococcal infections [28]. In particular, it was found that the maintenance of the total dalbavancin concentration ≥ 4.02 or 8.04 mg/L over time could grant a ≥90% likelihood of achieving an optimal pharmacodynamic target attainment against staphylococci with an MIC up to the MIC_90_ or the EUCAST clinical breakpoint of susceptibility for dalbavancin, respectively [28].

The main objective of this work was to describe the clinical outcome and safety of the sequential treatment with dalbavancin in a retrospective cohort of patient with spondylodiscitis caused by MRSA, and who were managed in the outpatient setting, after a short admission time. 

## 2. Results

Fifteen patients with spondylodiscitis by MRSA were included in the study. Table 1 summarizes all the clinical and laboratory characteristics of the studied patients. Median age was of 67.9 years (interquartile range, IQR: 57.4–78.5); while the main gender was the female one (8/15, 53.3%). The risk factors for spondylodiscitis were in most cases having had a previous surgery and having had a previous infection which was caused by MRSA in 6/15 (40%) of them and 3/15 (20%) of them, respectively. Importantly, overall, 11/15 (73.3%) patients have been hospitalized over the previous six months. Apart from having had previous surgery or infections, 10/15 (66.6%) patients had one or more risk factors for spondylodiscitis. Five patients (33.3%) presented more than 2 comorbidities, and the most common comorbidity was hypertension (8/15, 53.3%). Three patients had III stage chronic kidney diseases, CKD, (estimated glomerular filtration rate before starting dalbavacin were 55, 58, and 51 ml/minute for patient #5, #11, and #12, respectively). At the clinical presentation, 100% of the patients reported back pain, while a fever was present only in two cases (13.3%). Two patients (13.3%), patient #1 and patient #5, reported also right leg pain. Just one patient (patient #15) disclosed the inability to remain seated due to pain, and the localization of the infection was indeed to the sacral vertebrae. The spondylodiscitis was localized in 13/15 (86.6%) cases at the lumbar level. In three of the cases (20%), a paravertebral abscess was present (patient #1, #4, and #5). In 9/15 (60%) cases, the MRSA grew also in blood cultures. All the patients received an in-hospital intravenous treatment with vancomycin 25 mg/kg as a loading dose, which was followed by 15 mg/twice daily, and this was adjusted according to their kidney function for a median length of 14 (IQR:13–17) days. Before the availability of the microbiological culture, in all cases, vancomycin was associated with ceftriaxone. All our patients received their first dalbavancin dose as an inpatient immediately prior to discharge. The median number of dalbavancin doses was 4 (IQR: 3–4). None of the patient maintained a vascular access. The dalbavancin was administered in the outpatient setting through temporary peripheral venous accesses. Dalbavancin was used as monotherapy in 7 cases (46.7%), while in the remaining cases, it was associated with oral agents (moxifloxacin in 6/15, 40%; cotrimoxazole in 2/15, 13.3%). All the patients reported a clinical cure, except for an 80-year-old woman who is still on chronic suppressive treatment with dalbavancin for spondylodiscitis with infection of the vertebral stabilizing plates, with there being no surgical treatment option (due to age and multiple comorbidities). As for the secondary endpoint, all the patients were managed in an outpatient setting, and none of them needed either to be re-hospitalized or to have access to an emergency department for any reasons. The blood tests (full blood count, liver function tests, and creatinine values) remained stable and within the normal range during the antibiotic course and during follow-up. None of the patients developed serious treatment-related adverse events. In the patients with CKD, we did not detect alterations of their renal function and the dalbavacin dose adjustment was not necessary. 

The TDM for dalbavancin was performed in two patients (cases #3 and #4) who received four and nine dalbavancin TDMs during treatment, respectively. In patient #3, three out of four TDM determinations were above the threshold of 8.04 mg/L, ranging from 5.7 mg/L to 15.2 mg/L. In patient #4, all the dalbavancin TDMs were above the threshold of 8.04 mg/L, ranging from 12.6 mg/L to 53.2 mg/L.

## 3. Discussion

Spondylodiscitis is a complex disease, characterized by an infection of the vertebrae and the intervertebral disk space [29]. The severity of the infection is multifactorial, depending on the pathogenicity and virulence of the infectious agent, lesion location, systemic involvement, and whether or not the infection originated from other organs (e.g., a cardiac, gastrointestinal, or odontostomatological origin) [29,30]. Even though the improvements in surgical and radiological techniques as well as in antimicrobial therapies definitely changed the patient’s prognosis, the disease is still challenging with a high percentage of complications (16–32%), such as chronic pain, neurological disability, and a significant reduction in the quality of life [19].

In this study, we present our experience with dalbavancin for MRSA spondylodiscitis as a consolidation therapy, following a 2-week course of in-hospital antibiotic therapy. In our cohort, all cases but one, had a favourable outcome, both in the clinical and microbiological terms. As previously reported, in our patients, the most common symptom at the point of presentation was back pain, and the most common localization involved the lumbosacral region [16]. Importantly, at the last observation, all the patients were able to return to their normal daily activity, without experiencing any neurological complications. The microbiological cultures yielded the presence of MRSA in all cases, confirming the crucial role of the extensive microbiological work-up in the rapid initiation of a target therapy. Almost all the patients had risk factors for an MRSA infection (40% had previous surgery, 20% had a previous infection that was caused by MRSA, and 73.3% of the patients had been hospitalized over the previous 6 months). It is likely that the early identification of the causative organism may have played a role in the excellent outcome of the disease, avoiding a broad-spectrum empirical antibiotic therapy, and the subsequent risk of infectious complication such as *Clostridioides difficile* syndrome [31]. Indeed, *Staphylococcus aureus* remains the most frequently etiologic agent, accounting for 20–84% of pyogenic spondylodiscitis cases [15,32]. The yield of a *Staphylococcus aureus*-positive culture is of particular concern for at least two reasons: first, staphylococci produce many virulence factors, including adhesins, cytolytic toxins, immune-evasion factors, superantigens, and antioxidant systems, thereby making its treatment more troublesome [33]; second, the methicillin-resistance may have implications of lengthening the disease due to the poor tissue penetration of vancomycin [34]. Indeed, dalbavancin has a good penetration in bone tissue and a high efficacy against biofilms [35,36]. Dunne et al. determined the concentrations of it in synovial fluid and tissue after 14 days of a single infusion of 1000 mg of dalbavancin to be 15.9 μg/mL and 6.2 μg/mL, respectively, which are both above the MIC90 of dalbavancin for staphylococci [37]. Unfortunately, dalbavancin susceptibility testing were not available in our lab. However, it has been reported that using vancomycin as a surrogate drug in the same class to predict dalbavancin susceptibility has a predictive accuracy that is from 99.98% to 100.0% [38].

Notably, a proof-of-concept study recently highlighted the potential role that the TDM may have in giving real-time feedback about the estimated duration of the optimal treatment of a staphylococcal osteoarticular infection with dalbavancin in each single patient [28]. In the two cases for whom TDM was performed, we found that more than 90% determinations were above the threshold of 8.04 mg/L, thereby ensuring a very high probability of achieving an optimal pharmacodynamic target attainment against *Staphylococci* with a MIC up to the clinical breakpoint [28]. Therefore, this finding could suggest the potential remarkable role of the TDM in supporting clinicians in these challenging infections, thus allowing us to perform the most conservative scenario of the outpatient clinic. However, additional studies are needed to confirm our hypothesis. 

In our patients, vancomycin was initially used as first-line therapy (which is congruent with the guideline recommendations about the standard of care therapy) for a duration of about two weeks, and the dalbavancin was introduce in order to allow an early discharge. This also contributed to making this small cohort even more homogeneous. In addition, while optimal dosing and regimens for the off-label use of dalbavancin remain unknown, in our cohort dalbavancin was administered through a monthly scheduled infusion of 1500 mg, for a median duration of 12 weeks (for an overall amount of 4 doses). In our patients, dalbavancin was prescribed as a monotherapy in seven patients, whereas in the remaining eight it was associated with other antibiotics. To date, many different regimens have been tested for the treatment of spondylodiscitis, and the overall number of patients that are treated with dalbavancin is low [26,27]. Further, larger, and well-designed studies are needed to guide the selection of the appropriate agents and the duration of the antibiotic therapy. Moreover, the role of association with a possible oral treatment needs to be clarified.

Dalbavancin was well tolerated, and no drug discontinuations were observed, thereby confirming the safety profile of this long-acting drug also in a frail and aging population. Lastly, this experience underlines the role of dalbavancin as a cost-saving strategy primarily through the observations of early discharges and a reduction in length of the hospital stays. The advantages are not only in terms of costs, as demonstrated in some budget-impact analyses [10], but above all, in reducing the risk of acquiring nosocomial-acquired infections by providing an appropriate therapy when the long-term IV access for outpatient antibiotic administration is not feasible or should be avoided, and in improving the quality of life and mobility of the patients who could be safely monitored and followed-up in the outpatient’s setting [11].

Even if this is the first study reporting the off-label use of dalbavancin in a cohort of patients with spondylodiscitis by MRSA, our study conclusions are limited by the retrospective design, the lack of a control group, the lack of a TDM for all of the subjects, and the small number of patients.

## 4. Materials and Methods

In this study, we included all the patients who were older than 18 years of age, with documented spondylodiscitis by MRSA and who received treatment with at least one dose of dalbavancin (since it was available in our centre) as a sequential treatment at the Infectious Diseases Division of Padua University Hospital, Italy from January 2018 to January 2021. The microbiological diagnosis was made by the identification of the pathogen through a CT-scan-guided biopsy and blood cultures. Pending the microbiological results, an empirical treatment with vancomycin and ceftriaxone was administered to all the patients according to a local protocol that considers both the local epidemiology and individual risk factors. The treatment with dalbavancin was performed at an off-label dosage regimen of 1500 mg given on Days 1 and 8, which was followed by 1500 mg every 28–35 days. This dosing indeed provided a good plasma–drug concentration for 4–5 weeks according to the Monte Carlo simulation [39]. The dalbavancin dosage (an infusion of 1500 mg at day 1, which was followed by an infusion of 1500 mg at day 8) was selected according to the results from a randomized clinical trial [23] that showed that there was no difference in terms of the clinical cure between dalbavancin and vancomycin plus oral therapy in adult patients who were affected by osteomyelitis. Additionally, a population PK model that was reported that this dalbavancin regimen may ensure efficacy against both MSSA and MRSA for up to 5 weeks in patients with osteoarticular infections [39]. The need for further doses was established by the clinical indications.

The demographic, clinical and laboratory parameters, antibiotic therapy dosages and durations, safety outcomes, hospital length of stay, discharge disposition, and information about possible hospitalizations/access to emergency rooms were retrieved from the electronic health records. The primary endpoint was defined as the clinical cure. The clinical cure was defined by the resolution of all of the clinical signs and the symptoms of infection, an improvement of the radiological findings, and the persistent normalization of the C-reactive protein (CRP) values (i.e., values of CRP < 5 mg/L), no microbiological relapse during the follow-up period of 6 months after the end of treatment, and no need of switching to other antibiotic therapies for the patients who were initially treated with dalbavancin. Data on the treatment failure, adverse events, and toxicities (which were retrieved by medical records), the need to be hospitalized or access an emergency department for any reasons after the initiation of dalbavancin were also collected. In some patients, we performed a TDM of dalbavancin at the Clinical Pharmacology Unit of the IRCCS Azienda Ospedaliero-Universitaria di Bologna by means of a validated liquid chromatography-tandem mass spectrometry analytic method [40].

## 5. Conclusions

In conclusion, the results from our unique, homogenous, even if small, cohort demonstrated that dalbavancin can be a safe and effective option as a sequential treatment in patients with serious infections, such as spondylodiscitis, who require a prolonged antibiotic therapy. The implementation of a long-acting antibiotic treatment in the outpatient setting for patients with serious infections who are clinically stable should be desirable to improve their quality of life. Moreover, this option enabled the significant reduction both of length of stay and related resources. Further studies are necessary to confirm our results.

## Figures and Tables

**Table 1:** Full description of patient’s characteristics.

Patient ID	Gender	Age	Comorbidities	Symptoms at Presentation	CRP, mg/L * at the Baseline (Before Starting DAL)	Allergy	Previous Antibiotic Agent	Risk Factors	Localization	Reasons for DAL	Number of Dalbavancin Administration	Associated Oral Treatment	DAL Toxicities	Outcome
1	M	72.4	Hypertension, dyslipidaemia	Back pain, fever, right leg pain	132	None	Vancomycin	Previous MRSA infection	L2-L3-L4	Early discharge	4	Cotrimoxazole	None	Clinical cure
2	M	66.4	Hypertension, dyslipidaemia	Back pain	64	None	Vancomycin	Previous MRSA infection	D9-D12	Early discharge	4	Cotrimoxazole	None	Clinical cure
3	F °	57.8	Metastatic cancer, hypothyroidism, hypertension	Back pain	119	None	Vancomycin	Previous Surgery	L3-L4	Early discharge	5	Moxifloxacin	None	Clinical cure
4	F °	82.1	Hypertension	Back pain	180	None	Vancomycin	Previous Surgery	L5-S1-S2	Early discharge on patient’s request	14	None	None	Still ongoing/suppressive treatment
5	M	87.5	CKD, Dyslipidaemia, Diabetes	Back pain, fever, right leg pain	160	None	Vancomycin	Previous surgery	L4-S1	Early discharge	5	None	None	Clinical cure
6	F	46.8	None	Back pain	45	None	Vancomycin	IVDU	L3-L5	Early discharge	3	Moxifloxacin	None	Clinical cure
7	F	59.9	None	Back pain	97	None	Vancomyci, daptomycin	Not known	L3-L5	Early discharge	4	Moxifloxacin	None	Clinical cure
8	M	67.9	Cancer, hypertension, dislypidaemia	Back pain	165	None	Vancomycin	Previous surgery	L1-L2	Early discharge	4	Moxifloxacin	None	Clinical cure
9	F	68	None	Back pain	84	Penicillin and Fluoroquinolones	Vancomycin + rifampin	Previous surgery	L1-L2-L3	Early discharge	3	None	None	Clinical cure
10	F	75	Obesity	Back pain	20	None	Vancomycin	Previous surgery	L2-L3-L4	Early discharge	3	None	None	Clinical cure
11	F	94.4	CKD, Hypertension, Dementia	Back pain	60	None	Vancomycin	Not known	L4-L5-S1	Early discharge	3	None	None	Clinical cure
12	F	83.6	CKD, Diabetes, Hypertension	Back pain	25	None	Vancomycin	Previous MRSA infection	L2-L3	Early discharge	3	None	None	Clinical cure
13	M	50.5	None	Back pain	35	None	Vancomycin	Not Known	L1-L2-L3-L4	Early discharge	3	Moxifloxacin	None	Clinical cure
14	M	57	Depression, hypertension	Back pain	54	None	Vancomycin	Not Known	L4-L5	Early discharge	3	Moxifloxacin	None	Clinical cure
15	M	38.7	Depression	Back pain, difficult to remain sited	175	None	Vancomycin	IVDU	L5-S1-S2	Early discharge	4	None	None	Clinical cure

Legend to Table 1. F = female, M = male, CKD = chronic kidney disease, DAL = dalbavancin, IVDU = intravenous drug use, MRSA = *methicillin-resistant Staphylococcus aureus*, D = dorsal, L = lumbar, S = sacral, CRP = C reactive protein, * = mg/L, normal value < 5 mg/L, ° = patients with TDM monitoring available.

## Data Availability

The data that support the findings of this study are available on request to the corresponding author.
